# La Recherche du Temps Perdu: Timing in Somatosensation. Commentary: Somatosensation in the Brain: A Theoretical Re-evaluation and a New Model

**DOI:** 10.3389/fnsys.2020.597755

**Published:** 2020-11-12

**Authors:** Maria Del Vecchio, Pietro Avanzini

**Affiliations:** Consiglio Nazionale delle Ricerche, Istituto di Neuroscienze, Parma, Italy

**Keywords:** timing, four-dimensional, secondary somatosensory cortex (SII), touch, stereo-EEG (SEEG)

## Introduction

Somatosensation is a crucial component in our comprehension of the external world, not limited to the perception of shapes, temperature or pain, but also crucial for emotional and social interactions (Keysers et al., 2010). Recently, de Haan and Dijkerman (2020) have reviewed the cortical structures involved in somatosensation, and proposed a model in which core somatosensory regions (thalamus, primary and secondary somatosensory areas, responsible of basic somatosensory processing) would constitute the so called “cylinder block,” in turn informing a highly interconnected network subserving five high-order functions: haptic object recognition and memory, body perception, body ownership, affective processing, and action. Given their complexity and multimodal nature, such networks are largely overlapping in multiple processes, and we propose that this requires the complement of a fourth, *temporal* dimension to disambiguate, or associate, different functions. Most of the reviewed studies in de Haan and Dijkerman (2020) have been conducted with neuroimaging techniques, thus labeling areas as *active* following comparisons against the baseline or between different experimental conditions, but returning a static picture of the investigated function. Activation *per se*, however, might be an inefficient measure to disentangle the specific contribution of a cortical area within such complex functions. Moreover, despite adding relevant information about the causality of investigated regions, lesion studies also provide a similarly static outcome, failing to highlight dynamic signatures of somatosensory processing. This has led to the lack of mechanistic insights about the propagation of the information throughout such a highly distributed and interacting brain circuitry. As acknowledged in de Haan and Dijkerman (2020), however, dynamics represents a key issue when investigating somatosensation, without which its representation may be deceptive.

## Temporal Features Within Tactile Responses

Collecting human intracerebral recordings over 100 patients, Avanzini et al. (2016) reconstructed a four-dimensional picture of the brain activity following a passive tactile stimulation (i.e., contralateral median nerve). Despite the simplicity of the stimulus and the complete absence of cognitive tasks administered during the experimental session, activated regions were not limited to areas devoted to the encoding of tactile stimuli (e.g., SI and SII), extending also to fronto-parietal circuits subserving action execution and to insular cortex, involved in social touch and body ownership (Jenkinson et al., 2020).

Despite its richness, the sole spatial mapping was not sufficient to distinguish brain regions mediating low- and high-order functions. Rather, the investigation of the time-course of responsiveness represented the pivotal factor to operate such distinction, complementing the characterization of the somatosensory system with its dynamical behavior (de Lafuente and Romo, 2006). In Avanzini et al. (2016), authors clustered the gamma-band profiles of responsiveness following contralateral median nerve stimulation, finding two main patterns: *phasic* and *tonic* (see Figures 5–7 in Avanzini et al., 2018). *Phasic* responses (early, high-amplitude and short-lasting) subserve tactile encoding and proprioception, representing the core signature of the stimulus processing. *Tonic* responses (later, lower amplitude, long-lasting), instead, which are bilateral (Del Vecchio et al., 2019) and non-somatotopically arranged (Avanzini et al., 2018), pertain to areas involved in higher-order functions: haptic object recognition and tactile memory in bilateral SII (Ishida et al., 2013), social and affective touch in posterior insula (Kirsch et al., 2020), somatosensory processing for action execution (centro-parietal operculum) (Ishida et al., 2013).

Interestingly, such distinction was not dichotomic. This became evident in SII, which presented a *tonic* time-course coherently with the whole perisylvian region, but accompanied also by a *phasic*, component, suggesting an involvement of this area in multiple stages of the somatosensory processing. Thus, multiple functions can overlap in time and/or space, as the majority of neurons have multidimensional response properties, and their ensemble activity generates representations in a high-dimensional space (Gothard, 2020). In this framework, temporal features of response may become a fundamental discriminator to identify and distinguish among these functions (Avanzini et al., 2016).

## Temporal Similarity Suggests Functional Similarity

The relevance of temporal features in somatosensation becomes even stronger when considering the neural responses to complex tasks. Area SII, a node common to all the five higher-order networks (de Haan and Dijkerman, 2020), represents a highly explicative case. In a tactile frequency discrimination task, neuronal firing in SII is not phase-locked to the stimulus (contrary to SI and VPL thalamic nucleus), but rather the responses in SII are long-lasting, spanning intervals of hundreds of milliseconds after the stimulus delivery. A similar pattern was observed in frontal areas, but with a significantly longer latency (45 vs. 100 ms) (Romo and Rossi-Pool, 2020). The presence of such a sensory transformation in SII (Romo and Rossi-Pool, 2020) was entirely based on the timing of neuronal reactivity, and contributed fundamental knowledge about texture encoding, a key component of object recognition.

Likewise, a recent stereo-EEG study highlighted the value of temporal information as the factor that can disentangle the different roles played by SII in a complex and naturalistic task involving manipulative action execution and observation. In fact, while both single neuron recordings in macaques (Hihara et al., 2015) and neuroimaging studies (Ferri et al., 2015; Sharma et al., 2018) reported an *activation* of SII during the observation of actions, all these studies failed to indicate the role of such a visually-driven activation in a primarily somatosensory area. In a recent study using intracranial recordings (Del Vecchio et al., 2020), authors investigated the temporal pattern of responsiveness in somatosensory areas during an ecological reach-to-grasp and manipulate paradigm, revealing for SII a superimposable time course for both executed and observed actions, while SI activation was strictly confined to action execution. The overlap between the two temporal courses proved how SII is able to instantiate a representation of the observed motor act if this implies haptic exploration. This mirror-like response was revealed not just by the shared activation, but rather by the similarity between the temporal profiles exhibited during the two conditions, indicating that two completely different inputs determined convergent representations in SII.

A principle similarly inspired by co-activation of different brain areas is at the basis of the so-called functional connectivity, often used to date in both neuroimaging (Blatow et al., 2007) and electrophysiology (Auksztulewicz et al., 2012) to elucidate the network interactions specific for a given experimental condition.

## Conclusions

As highly interconnected as the somatosensory system is, time is a fundamental and non-negligible feature that allows characterizing and separating the contributions of different brain areas to complex functions. The rich information contained in somatosensory responses dynamicity advocates for a four-dimensional, time-wise representation of somatosensation. In particular, incorporating the temporal aspects into models of somatosensation could move the neural correlates from large and highly overlapping networks to smaller circuits with a dynamics specific for each of the investigated functions.

## Author Contributions

MD and PA conceived, drafted, and finalized the manuscript. Both authors contributed to the article and approved the submitted version.

## Conflict of Interest

The authors declare that the research was conducted in the absence of any commercial or financial relationships that could be construed as a potential conflict of interest.
